# Trajectories and predictors of self-care from pre-discharge to 12 months after discharge in people with spinal cord injury: a longitudinal study using growth mixture modeling

**DOI:** 10.3389/fnins.2026.1817190

**Published:** 2026-06-09

**Authors:** Ying Shen, Yuanyuan Su, Pan Luo, Yan Zhang, Qunqiang Wu, Xingzhi Lv, Yan Song

**Affiliations:** Department of Rehabilitation, Tangdu Hospital, The Fourth Military Medical University, Xian, China

**Keywords:** growth mixture model, longitudinal study, self-care, spinal cord injuries, trajectory

## Abstract

**Introduction:**

Self-care is essential for preventing secondary complications and supporting community reintegration after spinal cord injury (SCI). However, evidence is limited on how self-care changes over time after discharge and whether distinct subgroups exhibit different trajectories.

**Methods:**

Using convenience sampling, 220 patients with SCI were recruited from a tertiary hospital in northern China between August 2023 and December 2024. Self-care was measured with the Self-Care in Spinal Cord Injuries Inventory (SC-SCII) at pre-discharge baseline and at 1, 3, 6, and 12 months after discharge. Baseline mental health literacy and perceived social support were assessed using the Multicomponent Mental Health Literacy Scale (MMHL) and the Perceived Social Support Scale (PSSS). Repeated-measures ANOVA was used to describe the population-average time trend, and growth mixture modeling (GMM) was used as the primary person-centered analysis to identify latent trajectory classes. Multinomial logistic regression examined predictors of class membership.

**Results:**

A total of 209 patients completed all assessments (attrition rate: 5.0%). Mean SC-SCII scores peaked at 1 month post-discharge and then declined gradually over 12 months (*F* = 25.965, *p* < 0.001). GMM identified three distinct self-care trajectories: low-level decreasing (31.1%), moderate-level stable (39.7%), and high-level increasing (29.2%), with high classification accuracy (entropy = 0.965). Sex, educational level, mental health literacy, and perceived social support were associated with trajectory membership.

**Conclusion:**

Self-care trajectories after SCI are heterogeneous, and early post-discharge improvements may be transient for many individuals. The identified trajectories provide preliminary evidence for developing future prediction tools and trajectory-informed transitional nursing interventions.

## 1 Introduction

Spinal cord injury (SCI) refers to damage to the structure or function of the spinal cord caused by direct or indirect external factors, often resulting in severe motor, sensory, and autonomic dysfunction (Anjum et al., 2020). It is estimated that over 20.6 million people live with SCI worldwide, with approximately 900,000 new cases annually (Ding et al., 2022). As a profoundly disabling condition, SCI can have devastating impacts on physical health, mental well-being, social relationships, and employment (Budd et al., 2022), and has become a major public health challenge. Individuals with SCI often experience poorer mental health and quality of life (Sanguinetti et al., 2022), and incur a higher financial burden than the general population. For example, an investigation from Ontario, Canada reported a lifetime cost of approximately $336,000 per person, with higher costs among those with high-level injuries (Chan et al., 2019). Epidemiological studies indicate that the peak age of SCI onset varies across regions but is most commonly between 30 and 60 years; in China, an estimated 3.74 million people live with SCI, and around 70% are middle-aged or young adults (Hu et al., 2023). After injury, limitations in activities of daily living and risks of complications such as urinary tract infection, pressure injury, and deep vein thrombosis are common (Bhadra et al., 2025). Proactive self-care (e.g., pressure relief, skin checks, and bladder and bowel management) and effective self-care strategies are therefore essential to reduce preventable secondary conditions, support community participation, and improve recovery and prognosis (Kelly et al., 2022; Kurze et al., 2022; Piatt et al., 2016).

Self-care encompasses the behaviors and decisions patients make to manage their symptoms, treatments, physiological and psychosocial effects, and lifestyle adjustments (Barlow et al., 2002). Effective self-care is associated with better quality of life and health outcomes, and with reduced health service use (Benzo et al., 2016). Self-care among people with SCI is influenced by multiple factors, including demographic characteristics, disease-related factors, and psychosocial factors (Qama et al., 2025). Furthermore, self-care may change over time, with distinct developmental trajectories across subgroups. Therefore, identifying the dynamic trajectories of self-care and clarifying its key influencing factors in SCI is crucial for developing strategies to mitigate its adverse outcomes in this vulnerable population.

However, current evidence comes mainly from cross-sectional designs (van Diemen et al., 2018; McIntyre et al., 2020) and qualitative descriptive study (Munce et al., 2014), which cannot adequately capture within-person change, between-person heterogeneity, or the long-term evolution of self-care after discharge. These studies often treat individuals with SCI as a homogeneous group, although clinical experience suggests that patients may follow different recovery and adaptation patterns. Growth mixture modeling (GMM) is a person-centered longitudinal method that can identify latent subgroups with distinct developmental trajectories and may provide a basis for designing more targeted nursing follow-up strategies (van de Schoot, 2015). Therefore, this study aimed to (1) describe changes in self-care from pre-discharge baseline to 12 months after discharge among people with SCI, (2) identify latent trajectory classes of self-care using GMM, and (3) examine predictors of trajectory class membership.

## 2 Methods

### 2.1 Study design

A longitudinal observational study design was used. Baseline assessment was conducted before discharge, and follow-up assessments were conducted at 1, 3, 6, and 12 months after discharge.

### 2.2 Participants and data collection

**Figure 1:**
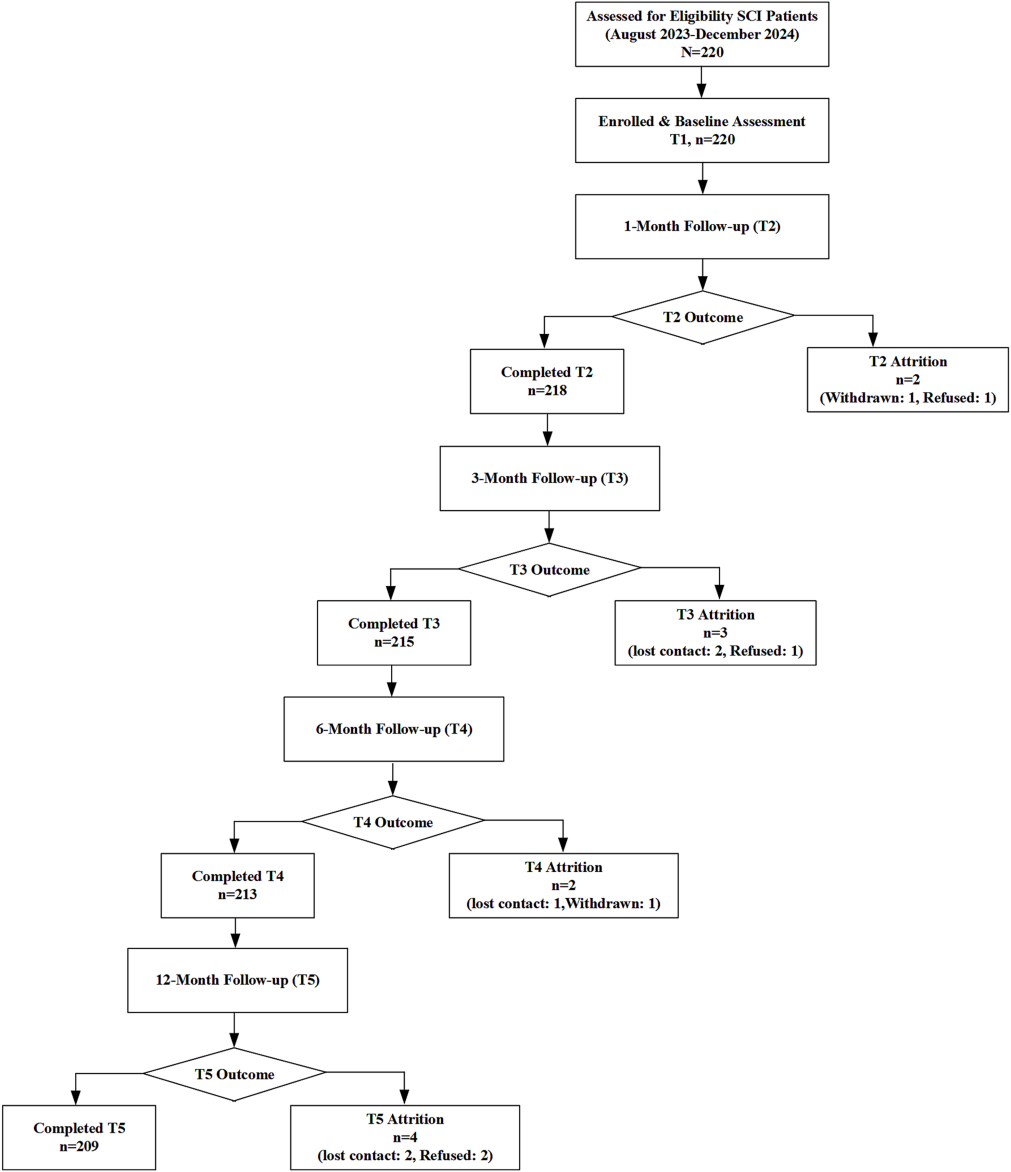
Flowchart of participant follow-up.

This study used convenience sampling to recruit inpatients with SCI from a tertiary hospital in northern China between August 2023 and December 2024. Inclusion criteria were: (1) SCI confirmed by magnetic resonance imaging; (2) classified as grade A-E according to the American Spinal Injury Association (ASIA) impairment scale (Roberts et al., 2017); (3) clear consciousness and able to communicate normally; and (4) provision of written informed consent. Exclusion criteria were severe organ dysfunction, current or previous malignant tumors, and a history of psychiatric disorders. Regarding sample size, GMM analysis generally recommends at least 200 participants (Lampousi et al., 2021). In addition, we performed an *a priori* calculation using G*Power 3.1 for repeated-measures ANOVA (effect size *f* = 0.25, *α* = 0.05, power = 0.80, five measurements), which indicated a minimum sample size of 136. Allowing for 10% attrition, 220 participants were enrolled. During follow-up, 11 participants were lost to follow-up (five could not be contacted, four refused, and two withdrew), yielding an attrition rate of 5.0% (Figure 1). Ultimately, 209 patients completed all assessments. Baseline characteristics were compared between completers and non-completers, and no statistically significant differences were observed in age, sex, education level, injury level, ASIA grade, MMHL score, and PSSS score (all *p* > 0.05).

### 2.3 Measures

#### 2.3.1 General demographic questionnaire

This questionnaire was developed by the research team based on a literature review and comprised two components: sociodemographic information and disease-related information. Sociodemographic information included age, sex, education level, marital status, living status, and per-capita monthly family income. Disease-related information included disease duration, neurological level of injury, and ASIA impairment grade.

#### 2.3.2 Self-care

The Self-Care in Spinal Cord Injuries Inventory (SC-SCII) developed by Conti et al. (2021), is used to assess self-care behaviors and self-care-related self-efficacy among individuals with SCI. Our team translated the instrument into Chinese and validated it (Wang et al., 2025). The scale contains 35 items across four domains: self-care maintenance (12 items), self-care monitoring (8 items), self-care management (8 items), and self-care self-efficacy (7 items). Items are rated on a 5-point Likert scale reflecting the frequency of behaviors (0 = “never” to 4 = “always”). Higher scores indicate better self-care. The Cronbach’s alpha of the Chinese version was 0.820.

#### 2.3.3 Multicomponent mental health literacy

The Multicomponent Mental Health Literacy Scale (MMHL) was developed by Jung et al. (2016) and validated in Chinese by Ming et al. (2021). It was used to measure mental health literacy. The scale includes 26 items across three dimensions: mental health knowledge (10 items), beliefs (8 items), and resources (4 items). The MMHL is scored dichotomously. For the knowledge and beliefs dimensions, items use a 5-point Likert format with an additional “do not know” option. In the knowledge dimension, “strongly agree” and “agree” are scored 1; in the beliefs dimension, “strongly disagree” and “disagree” are scored 1; all other responses are scored 0. The resources dimension consists of yes/no items (“yes” = 1, “no” = 0). Higher total scores indicate higher mental health literacy. The Cronbach’s alpha in the Chinese version was 0.800.

#### 2.3.4 Social support

The Perceived Social Support Scale (PSSS), developed by Zimet et al. (1990) and introduced into China by Jiang (1999), was used to assess perceived social support. The scale includes 12 items across three dimensions: family support, friend support, and other support, with four items per dimension. Items are rated on a 7-point Likert scale (1 = “strongly disagree” to 7 = “strongly agree”), with higher scores indicating greater perceived social support. The Cronbach’s alpha of the Chinese version was 0.890.

### 2.4 Procedures for data collection

Questionnaire data were collected at five time points: pre-discharge baseline (T1) and 1 month (T2), 3 months (T3), 6 months (T4), and 12 months (T5) after discharge. Within 3 days of admission, general information was extracted from the medical record system. Eligible participants were screened according to the inclusion and exclusion criteria. After informed consent was obtained, baseline assessment was conducted before discharge. At T1, the general information questionnaire, SC-SCII, MMHL, and PSSS were administered; at T2-T5, follow-up assessments were conducted using the SC-SCII. To improve data completeness and reliability, two trained researchers independently checked questionnaire completeness. Participants completed the questionnaires by self-report, and all data were de-identified and kept confidential. After each assessment, the next follow-up was scheduled. A WeChat group was created, and two contact methods were recorded for each participant. Participants were reminded of follow-up visits through WeChat or telephone as needed.

### 2.5 Statistical analysis

Analyses were performed using SPSS 26.0 and Mplus 8.0. Continuous variables with a normal distribution were presented as mean ± SD and compared using ANOVA; non-normally distributed variables were summarized as median (P25, P75) and compared using the Kruskal–Wallis test. Categorical variables were presented as frequencies and percentages and compared using the chi-square test or Fisher’s exact test, as appropriate. The primary analyses were conducted among participants with complete SC-SCII data at all five time points (*n* = 209). Overall changes in SC-SCII scores across time points were examined using repeated-measures ANOVA. Mauchly’s test was used to assess sphericity; when the sphericity assumption was violated, Greenhouse–Geisser correction was applied. GMM was then used as the primary person-centered analysis to identify latent subgroups with distinct self-care trajectories. Time was coded as months since pre-discharge baseline (0, 1, 3, 6, and 12 months) to reflect the unequal spacing of assessments. Because the descriptive trend suggested a possible non-linear pattern, unconditional linear, quadratic, and piecewise growth functions were evaluated. For the piecewise model, the knot was placed at 1 month after discharge to distinguish the early transition period from the later follow-up period. Models with one to five latent classes were fitted. Model fit for the GMM was evaluated using: (1) the Akaike information criterion (AIC), Bayesian information criterion (BIC), and sample-size-adjusted BIC (aBIC), with smaller values indicating better fit; (2) entropy (range 0–1), with values closer to 1 indicating better classification accuracy; and (3) the Lo–Mendell–Rubin adjusted likelihood ratio test (LMR) and the bootstrap likelihood ratio test (BLRT), where *p* < 0.05 indicates that a model with k classes fits significantly better than a model with k-1 classes (Berlin et al., 2014; Kim, 2014). Results across indices were considered together to determine the optimal model. In Mplus, robust maximum likelihood estimation was used, with STARTS = 1,000 250 and STITERATIONS = 20. Multinomial logistic regression was used to identify factors associated with self-care trajectory class membership. Multicollinearity was assessed using variance inflation factors (VIFs), with VIF values <5 considered acceptable. The significance level was set at alpha = 0.05.

## 3 Results

### 3.1 Descriptive statistics

During the study period, five patients could not be contacted, four refused participation, and two withdrew. In total, 209 patients completed all five assessments and were included in the complete-case analyses. Of the 209 participants, 151 (72.2%) were male and 58 (27.8%) were female; the mean age was 45.66 ± 11.25 years. Regarding education level, 36 participants had primary school education or below, 76 had junior high school education, and 97 had technical secondary school education or above. A total of 143 participants were married, and 66 were unmarried or divorced. Twenty-two participants lived alone, and 187 lived with family members. Detailed characteristics are shown in Table 1.

**Table 1:** Univariate comparisons across self-care trajectory classes in patients with SCI (*N* = 209).

Variables	*n* (%)	low-level decreasing (*n* = 65)	moderate-level stable (*n* = 83)	high-level increasing (*n* = 61)	*F*/*χ*^2^/*H*	*p*
Age (years, $\bar{x}$ ± SD)		46.60 ± 11.52	45.49 ± 10.35	44.87 ± 12.20	0.385	0.681
Sex (*n*, %)					8.333	0.031
Male	151 (72.2)	47 (72.3)	64 (77.1)	40 (65.6)		
Female	58 (27.8)	18 (27.7)	19 (22.9)	21 (34.4)		
Education level (*n*, %)					11.385	0.002
Primary school and below	36 (17.2)	16 (24.6)	14 (16.9)	6 (9.8)		
Junior high school	76 (36.4)	28 (43.1)	34 (40.9)	14 (23.0)		
Technical secondary school and above	97 (46.4)	21 (32.3)	35 (42.2)	41 (67.2)		
Marital status (*n*, %)					6.625	0.157
Married	143 (68.4)	48 (73.9)	59 (71.1)	36 (59.0)		
Unmarried	32 (15.3)	6 (9.2)	15 (18.1)	11 (18.0)		
Divorced	34 (16.3)	11 (16.9)	9 (10.8)	14 (23.0)		
Living status (*n*, %)					7.797	0.099
Living alone	22 (10.5)	10 (15.4)	3 (3.6)	9 (14.8)		
Living with spouse or children	187 (89.5)	55 (84.6)	80 (96.4)	52 (85.2)		
Per capita monthly family income (*n*, %)					5.625	0.229
<3,000 yuan	63 (30.2)	25 (38.5)	23 (27.7)	15 (24.6)		
3,000–5,000 yuan	86 (41.1)	26 (40.0)	37 (44.6)	23 (37.7)		
>5,000 yuan	60 (28.7)	14 (21.5)	23 (27.7)	23 (37.7)		
Disease duration (Months, $\bar{x}$ ± SD)		19.71 ± 17.82	23.00 ± 15.32	20.85 ± 14.48	0.822	0.441
Spinal cord injury level (*n*, %)					2.955	0.565
Cervical	89 (42.6)	24 (36.9)	39 (47.0)	26 (42.6)		
Thoracic	81 (38.7)	28 (43.1)	27 (32.5)	26 (42.6)		
Lumbosacral	39 (18.7)	13 (20.0)	17 (20.5)	9 (14.8)		
ASIA classification (*n*, %)					0.046	0.977
Class A	40 (19.1)	13 (20.0)	14 (16.9)	13 (21.3)		
Class B	58 (27.7)	13 (20.0)	27 (32.5)	18 (29.5)		
Class C	43 (20.6)	16 (24.6)	16 (19.3)	11 (18.0)		
Class D	43 (20.6)	16 (24.6)	18 (21.7)	9 (14.8)		
Class E	25 (12.0)	7 (10.8)	8 (9.6)	10 (16.4)		
MMHL [points, M (P25, P75)]		8.0 (6.0, 9.0)	12.0 (10.0, 14.5)	16.0 (15.0, 17.0)	26.314	<0.001
PSSS (points, $\bar{x}$ ± SD)		39.83 ± 6.91	52.59 ± 7.63	60.51 ± 6.90	33.249	<0.001

### 3.2 Self-care scores at different time points among patients with SCI

**Figure 2:**
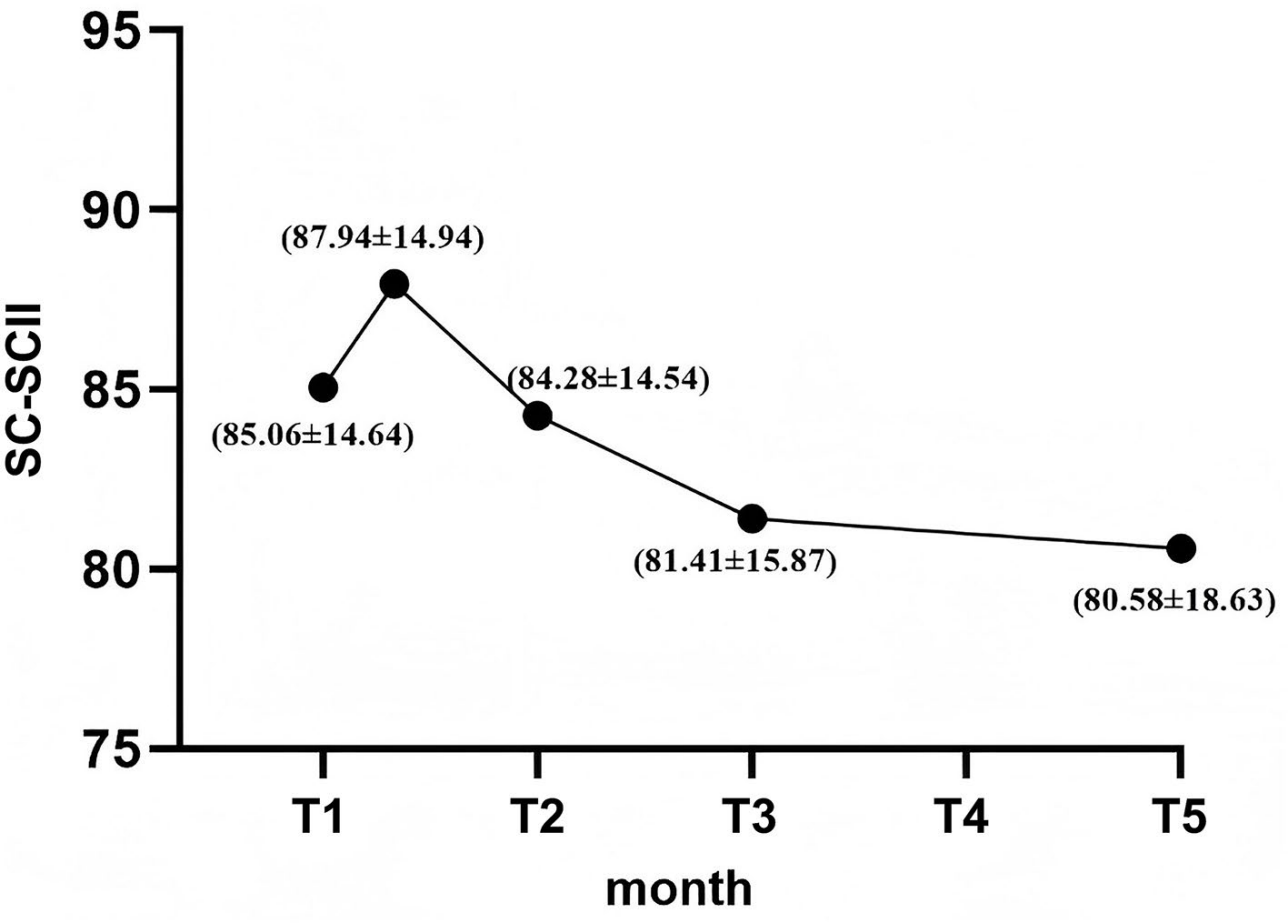
Observed mean SC-SCII scores at each assessment time point (T1–T5).

The mean SC-SCII scores were highest at 1 month after discharge and then declined overall through 12 months, with a slight rebound at 6 months (*F* = 25.965, *p* < 0.001; Figure 2). Mauchly’s test indicated that the sphericity assumption was violated (*W* = 0.721, chi-square = 67.842, *p* < 0.001); therefore, Greenhouse–Geisser correction was applied (epsilon = 0.782). The time effect remained statistically significant after correction (adjusted *p* < 0.001).

### 3.3 Latent classes of self-care change trajectories in SCI

GMM was used to fit the self-care trajectories of the 209 patients. Models with one to five classes were fitted, and the fit indices are presented in Table 2. Although information criteria continued to decrease as the number of classes increased, the LMR and BLRT results were significant for the two- and three-class models but not for the four- and five-class models. The three-class model also showed high entropy (0.965), adequate class sizes, and clinically interpretable trajectories. Linear, quadratic, and piecewise growth functions were examined because the descriptive trend suggested a possible peak-and-decline pattern. Compared with the linear and quadratic specifications, the three-class piecewise model showed better overall fit and clearer clinical interpretability, while maintaining stable convergence and replicated log-likelihood (Table 3). The final piecewise model used a knot at 1 month after discharge. Based on the overall evaluation, a three-class piecewise solution was considered the most appropriate representation of self-care change trajectories.

**Table 2:** Fit indices for one- to five-class piecewise GMM solutions.

Class	AIC	BIC	aBIC	LMR (*p*)	BLRT (*p*)	Entropy	Class probability (%)
1	8,954.481	8,987.905	8,956.219	–	–	–	
2	7,971.260	8,024.737	7,974.041	<0.001	<0.001	0.961	0.397/0.603
3	**7,449.452**	**7,522.984**	**7,453.276**	**<0.001**	**<0.001**	**0.965**	0.311/0.397/0.292
4	7,278.270	7,371.855	7,283.136	0.336	0.345	0.948	0.273/0.306/0.273/0.148
5	7,161.503	7,275.142	7,167.412	0.668	0.671	0.932	0.182/0.143/0.244/0.206/0.225

The final selected GMM model.

**Table 3:** Comparison of alternative three-class growth functions.

Growth form	Class	AIC	BIC	aBIC	Entropy	LMR (*p*)	BLRT (*p*)	Smallest class (%)
Linear	3	7,528.614	7,595.462	7,532.089	0.941	<0.001	<0.001	29.2
Quadratic	3	7,473.886	7,554.104	7,478.057	0.958	<0.001	<0.001	29.2
Piecewise	3	7,449.452	7,522.984	7,453.276	0.965	<0.001	<0.001	29.2

For the selected three-class piecewise GMM, the average posterior probabilities were high for all classes (C1 = 0.971, C2 = 0.954, and C3 = 0.968), supporting good classification quality (Table 4). The trajectory plot showed that C1 followed a low-level decreasing pattern, C2 showed a relatively stable moderate-level pattern, and C3 showed a high-level increasing pattern. The average posterior probabilities and class-specific piecewise growth parameters are shown in Table 4.

**Table 4:** Classification quality and piecewise growth parameters of the selected three-class GMM.

Class	*n* (%)	Average posterior probability	Intercept	Early slope	Late slope
C1	65 (31.1)	0.971	67.80	4.30	−1.25
C2	83 (39.7)	0.954	82.60	3.50	−0.40
C3	61 (29.2)	0.968	95.40	5.60	0.96

**Figure 3:**
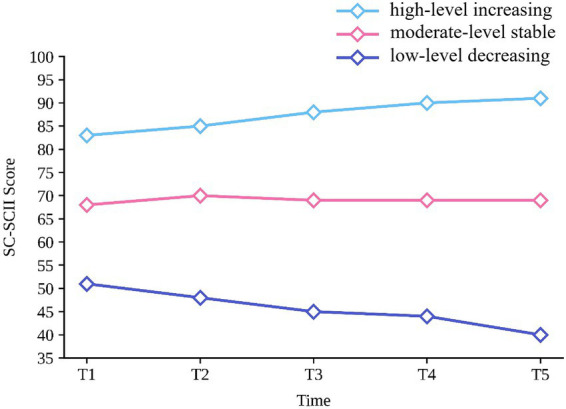
Latent class trajectory diagram of self-care in patients with SCI. Values represent model-estimated mean SC-SCII scores from the selected three-class piecewise GMM at pre-discharge baseline and 1, 3, 6, and 12 months after discharge.

Based on the initial levels of the three trajectories and the patterns of change in SC-SCII scores over time, the classes were named as follows: (1) Class 1: low-level decreasing group (C1; *n* = 65, 31.1%), characterized by a low initial score with a continued downward trend during follow-up; (2) Class 2: moderate-level stable group (C2; *n* = 83, 39.7%), characterized by a relatively higher initial score that remained stable over time; and (3) Class 3: high-level increasing group (C3; *n* = 61, 29.2%), characterized by the highest initial self-care score and a further increasing trend over time. See Figure 3.

### 3.4 Univariate analysis of the trajectory of self-care changes in SCI

Univariate analyses showed statistically significant differences among the trajectory classes in sex, education level, MMHL scores, and PSSS scores (*p* < 0.05), whereas age, marital status, living status, income, disease duration, neurological level of injury, and ASIA grade were not significantly different across classes (*p* > 0.05; Table 1).

### 3.5 Multivariable analysis of self-care trajectories in SCI

Trajectory class membership was used as the dependent variable, with C1 as the reference group in multinomial logistic regression. To avoid relying solely on univariate screening, we constructed a clinically informed multinomial logistic regression model based on prior literature and clinical relevance. Variables significant in univariate analyses, including sex, educational level, MMHL, and PSSS, were entered together with key demographic and injury-related covariates selected *a priori*, including age, disease duration, neurological level of injury, and ASIA impairment grade. Multinomial logistic regression results showed that higher education level, higher MMHL score, and higher PSSS score were associated with more favorable self-care trajectories. Female sex was significantly associated with membership in the high-level increasing group, but not in the moderate-level stable group. Age, disease duration, neurological level of injury, and ASIA grade were not independently associated with trajectory membership. Compared with C1, female participants had higher odds of being classified into C2 (OR = 1.56, 95% CI: 0.86–2.84) or C3 (OR = 1.95, 95% CI: 1.01–3.76). Higher education showed a graded association with more favorable trajectory membership. Each one-point increase in MMHL was associated with higher odds of C2 membership (OR = 1.15, 95% CI: 1.05–1.27) and C3 membership (OR = 1.27, 95% CI: 1.12–1.44), and each one-point increase in PSSS was associated with higher odds of C2 membership (OR = 1.04, 95% CI: 1.01–1.08) and C3 membership (OR = 1.06, 95% CI: 1.02–1.10; Table 5). Multicollinearity diagnostics showed acceptable collinearity, with VIFs ranging from 1.04 to 1.76.

**Table 5:** Multinomial logistic regression analysis of self-care trajectory membership among patients with SCI.

Class	Variables	B	SE	Wald *χ*^2^	*p*	OR (95% CI)
C2 vs. C1	Female	0.445	0.305	2.129	0.145	1.56 (0.86–2.84)
	Education level (Junior high school)	0.329	0.313	1.106	0.293	1.39 (0.75–2.56)
	Education level (Technical secondary school and above)	0.652	0.313	4.338	0.037	1.92 (1.04–3.55)
	MMHL	0.140	0.049	8.295	0.004	1.15 (1.05–1.27)
	PSSS	0.039	0.017	5.264	0.022	1.04 (1.01–1.08)
	Age	−0.010	0.015	0.422	0.516	0.99 (0.96–1.02)
	Disease duration	−0.010	0.010	0.951	0.330	0.99 (0.97–1.01)
	Neurological level (Thoracic)	−0.117	0.345	0.114	0.736	0.89 (0.45–1.74)
	Neurological level (Lumbosacral)	−0.041	0.421	0.009	0.923	0.96 (0.42–2.19)
	ASIA grade	0.068	0.111	0.370	0.543	1.07 (0.86–1.33)
C3 vs. C1	Female	0.668	0.335	3.966	0.046	1.95 (1.01–3.76)
	Education level (Junior high school)	0.615	0.314	3.828	0.049	1.85 (1.00–3.43)
	Education level (Technical secondary school and above)	0.990	0.329	9.021	0.003	2.69 (1.41–5.13)
	MMHL	0.239	0.064	13.899	<0.001	1.27 (1.12–1.44)
	PSSS	0.058	0.019	9.151	0.002	1.06 (1.02–1.10)
	Age	−0.020	0.018	1.241	0.265	0.98 (0.95–1.02)
	Disease duration	−0.020	0.013	2.433	0.119	0.98 (0.96–1.01)
	Neurological level (Thoracic)	0.049	0.371	0.017	0.895	1.05 (0.51–2.18)
	Neurological level (Lumbosacral)	−0.198	0.482	0.169	0.681	0.82 (0.32–2.12)
	ASIA grade	0.122	0.123	0.991	0.319	1.13 (0.89–1.44)

C1 = low-level decreasing group; C2 = moderate-level stable group; C3 = high-level increasing group. MMHL = Multicomponent Mental Health Literacy Scale; PSSS = Perceived Social Support Scale. Sex was coded as male = 0 and female = 1. Education level was coded with primary school and below as the reference category. Neurological level was coded with cervical injury as the reference category. ASIA grade was entered as an ordinal variable from A to E. Age, PSSS, MMHL and disease duration were entered as continuous variables.

## 4 Discussion

This longitudinal study examined changes in self-care from pre-discharge baseline to 12 months after discharge in patients with SCI. The SC-SCII score peaked 1 month after discharge and then gradually declined, which is broadly consistent with the decline in self-care ability reported by Jiang et al. (2021). This pattern may reflect a short supported transition window, during which patients still benefit from recent discharge education, rehabilitation routines, and intensive family attention. As patients return to complex home and community environments, the cumulative workload of secondary-complication prevention, skin care, and bladder/bowel programs may become more difficult to sustain, while access to professional follow-up and rehabilitation resources may be limited. A similar trend was seen in the study of LaVela et al. (2016), who found that patients with SCI gradually declined in self-care behaviors within 6 months after discharge, especially in daily care and complication prevention. Qualitative research also highlights that post-discharge barriers, such as fragmented services, environmental constraints, competing life demands, and diminished confidence over time, may erode adherence to self-care behaviors (Munce et al., 2014). These explanations should be interpreted as plausible mechanisms rather than causal findings, because service fragmentation, self-efficacy, and rehabilitation access were not directly measured in the present study. This finding suggests that the early post-discharge improvement may not predict longer-term self-care maintenance. Transitional support may therefore need to extend beyond discharge planning and include stepwise follow-up over 6–12 months, particularly after the early post-discharge period.

### 4.1 Three latent classes of self-care behavior trajectories among patients with SCI

Using GMM, we identified three distinct self-care trajectories: low-level decreasing (31.1%), moderate-level stable (39.7%), and high-level increasing (29.2%). This finding is consistent with the systematic review by Conti et al. (2022), which indicated that self-care behaviors in people with SCI are heterogeneous. Clinically, these findings suggest that relying only on average self-care scores may obscure important subgroup differences. The moderate-level stable group may represent patients who establish a workable routine but do not continue to improve, suggesting that maintenance and reinforcement strategies, such as booster sessions, barrier problem-solving, goal setting, and action planning, could be explored in future intervention studies (Lorig et al., 1999; Powers et al., 2020). The high-level increasing group suggests that some patients may gradually transform knowledge into habit and build mastery experiences over time. For this group, periodic reinforcement and empowerment-focused coaching may be sufficient, although this requires prospective validation. The low-level decreasing group, which accounted for nearly one-third of the cohort, is clinically concerning because persistently low and declining self-care may increase vulnerability to preventable complications, such as pressure injuries and urinary tract infections, and unplanned healthcare use (Verschueren et al., 2011). However, because class membership was identified retrospectively from longitudinal data, these classes should not be interpreted as a validated admission-screening tool. Rather, they provide a basis for future studies to develop and validate early prediction models and trajectory-informed intervention pathways.

Taken together, these findings support the hypothesis that one-size-fits-all discharge education may be insufficient for patients with SCI. Trajectory-based classification may help generate more precise hypotheses about which patients require intensive transitional support, which patients need maintenance-oriented reinforcement, and which patients may benefit from empowerment-focused follow-up. Future studies should prospectively test whether early clinical, psychosocial, and functional indicators can accurately assign new patients to likely trajectory patterns.

### 4.2 Predictors of self-care behavior trajectory membership among patients with SCI

Multivariable analyses indicated that sex, education level, MMHL, and PSSS were associated with trajectory membership. Being female and having higher education, higher MMHL, and higher PSSS were associated with greater likelihood of belonging to more favorable self-care trajectories.

#### 4.2.1 Sex

Female participants were more likely to exhibit favorable self-care trajectories. This finding is compatible with a large cross-national comparative study of 12,588 people with SCI across 22 countries (Bychkovska et al., 2024). This may be related to social gender roles, as women are usually given more home care responsibilities and thus have a higher level of learning and attention to care skills. Additionally, women may be more likely to express health needs, engage with educational resources and communication during care transitions, and use social support resources (Craven and Musselman, 2019). But these mechanisms were not directly tested in the current study. Therefore, rather than assuming uniform needs across sex, nursing assessment should focus on individual barriers to self-care engagement, communication preferences, and practical problem-solving needs during the early post-discharge period.

#### 4.2.2 Education level

Higher educational level was associated with membership in more favorable self-care trajectories. This is consistent with the review by Barlow et al. (2002), which suggested that education may support self-management by improving information processing, critical thinking, and communication with the healthcare system. Patients with higher education levels may be better able to understand complex medical information, translate it into daily care routines, seek trustworthy information, and solve practical self-care problems (Sørensen et al., 2015). For patients with lower education levels, education should be delivered using images, video demonstrations, simplified language, teach-back methods, repeated reinforcement, phone reminders, and simple checklists rather than text-heavy materials. Understanding should be assessed directly to ensure that information has been effectively transferred.

#### 4.2.3 Mental health literacy

MMHL was associated with trajectory membership. Mental health literacy refers to knowledge and beliefs that aid recognition, management, and prevention of mental disorders (Jorm et al., 1997). People with SCI commonly experience depression, anxiety, and suicidal ideation, which may undermine motivation, problem solving, and adherence to self-care routines (Betthauser et al., 2023). Higher mental health literacy may facilitate earlier recognition of psychological distress, timely help-seeking, use of adaptive coping strategies, and communication with clinicians, thereby supporting self-care maintenance. This finding suggests that mental health literacy screening and brief psychoeducation could be considered in future transitional care programs, especially for patients showing early signs of declining self-care.

#### 4.2.4 Perceived social support

Perceived social support was associated with trajectory membership, consistent with research emphasizing the role of family, peers, and accessible resources in sustaining self-care (Newsom et al., 2025). Social support may facilitate self-care through instrumental assistance, informational support, reminders, emotional encouragement, and confidence building (Graven and Grant, 2014). In this study, the high-level increasing group had the highest PSSS score, suggesting that a strong support network may help patients maintain self-care routines despite functional limitations. Nevertheless, social support, self-efficacy, caregiver availability, and rehabilitation access are likely interrelated, and causality cannot be inferred from the present observational design. A randomized trial demonstrated that a peer-led, telephone-based empowerment intervention improved health self-management outcomes in adults with chronic SCI (Houlihan et al., 2017). Digital and mobile health tools may further help maintain engagement when in-person follow-up is limited, and a systematic review summarized the potential of mobile health self-care support tools for SCI (Bernard et al., 2023). Future studies should examine whether family-centered discharge planning, caregiver training, peer support, and telehealth follow-up can alter unfavorable self-care trajectories.

### 4.3 Implications for nursing practice and research

This trajectory-based framework provides preliminary evidence that self-care after SCI is heterogeneous and may require different levels of transitional support. However, the trajectory classes in this study were identified retrospectively after observing five repeated measurements; therefore, they should not yet be used as a stand-alone tool for prospective clinical stratification at admission or discharge. A more appropriate implication is that these findings can guide future development of prediction models and intervention trials. In future studies, early indicators such as baseline SC-SCII score, mental health literacy, perceived social support, injury severity, functional independence, complications, and rehabilitation access could be combined to predict likely trajectory membership. If externally validated, such tools may help nurses identify patients who need intensive early follow-up, maintenance-oriented reinforcement, or empowerment-focused support.

## 5 Limitations

This study has several limitations. First, the sample size was modest, and the single-center convenience-sampling design may limit generalizability. Second, although the overall attrition rate was low, the primary analyses were based on complete cases; therefore, selection bias cannot be fully excluded, particularly if participants lost to follow-up differed systematically from completers. Third, although several injury-related variables were examined, important clinical and contextual factors, such as functional independence, secondary complications, rehabilitation intensity, rehabilitation accessibility, caregiver burden, and home environmental barriers, were not fully measured. These factors may confound or mediate the associations between psychosocial predictors and self-care trajectories. Fourth, latent class assignment involves model uncertainty, and the selected three-class solution may be affected by sample size, model specification, and potential overfitting. Fifth, self-care was assessed using self-reported measures, which may introduce recall and social desirability bias. Future multicenter studies with larger samples, richer clinical indicators, objective outcomes (e.g., pressure injury incidence, urinary tract infection episodes, rehospitalization), and external validation are needed to refine trajectory-informed nursing care pathways.

## 6 Conclusion

Self-care from pre-discharge baseline to 12 months after discharge did not follow a uniform trajectory among patients with SCI. Self-care increased at 1 month after discharge and then declined over the following months. Three distinct self-care trajectories were identified: low-level decreasing, moderate-level stable, and high-level increasing. Sex, educational level, mental health literacy, and perceived social support were associated with trajectory membership. These findings provide preliminary evidence for future development of validated prediction tools and trajectory-informed transitional care strategies.

## Data Availability

The raw data supporting the conclusions of this article will be made available by the authors, without undue reservation.
